# Literary Notes

**Published:** 1876-08

**Authors:** 


### LITERARY NOTICES.

*Appleton's Journal.* — This magazine, formerly issued as a weekly, is now published as a monthly. It is one of the largest and best of our literary monthlies; finely illustrated, and presenting an attractive table of contents each month. It is a fireside friend. The ladies always welcome it. Price $4 00 a year. D. Appleton & Co., New York.

*Lippincott's Illustrated Magazine* is one of the purest and best of the literary monthlies. It is always replete with the finest tales of fiction, and embellished with attractive illustrations. It is a welcome visitor to the family circle. Price $4 00 a year. J. B. Lippincott & Co., Philadelphia, Pa.

